# In Vitro Risk Assessment of Dental Acid Erosion Caused by Long-Term Exposure to Oral Liquid Bandages

**DOI:** 10.3390/dj12030070

**Published:** 2024-03-06

**Authors:** Ryouichi Satou, Naoki Sugihara

**Affiliations:** Department of Epidemiology and Public Health, Tokyo Dental College, Tokyo 101-0061, Japan; sugihara@tdc.ac.jp

**Keywords:** erosion, liquid bandage, enamel, dentin, demineralization, risk assessment

## Abstract

Oral mucosa inflammation can cause severe pain and interfere with eating, reducing quality of life. However, few options for self-care are available. An oral liquid bandage forms a protective film over the affected area. We aimed to assess the acid erosion risk when a newly developed oral liquid bandage (ORAPLA) is accidentally deposited on teeth and to examine the relative acid erosion risk at multiple time points of the maximum recommended duration of continuous use. ORAPLA was applied to both enamel and dentin blocks from 45 bovine anterior mandibular teeth, and an acid challenge was performed in a simulated oral cavity with artificial saliva, with one exposure cycle lasting 6 h. The enamel showed substantial defects and a decrease in Vickers hardness after nine cycles, with no change in surface roughness. Dentin showed an increase in parenchymal defects and surface roughness and a trend toward decreased Vickers hardness with increasing exposure time. We found no significant acid corrosion in enamel after up to nine times the upper limit of normal use time or in dentin after up to six times the upper limit. We conclude that the acid erosion risk due to accidental attachment to teeth is low, and in the human oral cavity with salivary buffering and remineralization, likely even lower.

## 1. Introduction

Acid erosion of teeth is generally understood as the dissolution of tooth structure resulting from chemical reactions that don’t involve microorganisms. Acidic drinks, foods, or medical substances with a pH lower than 5.5 can cause rapid demineralization of enamel, resulting in the loss of minerals such as calcium and phosphorus, which make up the structural components of hydroxyapatite [1]. Exposed dentin due to gingival recession has a high collagen content and a critical pH of 6.0–6.2, making it highly susceptible to acids [1]. Erosion caused by pharmaceuticals is caused by the habit of chewing acidic drug capsules with teeth or accidentally allowing acidic solutions or ointments to stick to the teeth and remain there for a long period of time [1]. In the case of long-term medication for treatment, a doctor’s judgment is required to stop taking the medication, which is difficult to perform at a dental clinic. Therefore, it is necessary to clarify in advance the effects of dental erosion caused by long-term exposure to pharmaceuticals.

Stomatitis, a severely painful condition caused by injury, inflammation, or contact with the oral mucosa, interferes with eating and communication and reduces the quality of life [2,3]. The oral mucosa is exposed to the outside world and is subjected to frequent physical, chemical, or thermal injuries that tend to recur in the same area. The most common types of stomatitis of the oral mucosa are aphthous and catarrhal stomatitis [2,4,5]. Aphthous stomatitis presents with characteristic symptoms, such as round or oval lesions featuring clearly defined edges, central necrosis covered by a yellow-grey membrane, and an inflamed ring around the lesion, indicative of peripheral inflammation [2,3,6,7]. Recurrent aphthous lesions are frequent in children and can interfere with normal feeding, swallowing, and speech [7,8]. Catarrhal stomatitis is observed in all age groups and is induced by physical stimuli. In clinical dentistry, stomatitis reportedly occurs when orthodontic appliances or dentures used in orthodontic treatment physically irritate mucous membranes, causing wounds and inflammation [2,9]. It can also be caused by bacterial growth-associated trauma, such as bites on the buccal mucosa or irritation from hot water or chemicals. The catarrhal form shows indistinct borders and edema, and a burning sensation, halitosis, and blunted taste are the main symptoms [3,10]. The section of the Japanese Orthodontic Association’s guidelines titled “Risks and Side Effects of Orthodontic Treatment” indicates that orthodontic appliances may cause discomfort, mouth ulcers, and pain due to tooth movement at the beginning of treatment [11]. However, the rapid turnover of the oral mucosal epithelium (6–12 days) allows for rapid wound healing and a good prognosis in many cases; therefore, the care of wounds and stomatitis on the oral mucosa is often neglected in general dental practice.

Managing inflammation of the oral mucosa is also a crucial aspect of ward care for individuals with systemic illnesses. Oral mucositis (OM) is an adverse event associated with chemotherapy and radiation therapy [12]. About 40% of patients who receive chemotherapy develop mucositis, and this percentage rises to around 90% for individuals with head and neck cancer who undergo both chemotherapy and radiation therapy [6]. OM induced by cancer treatment primarily manifests as erythema, atrophy, or ulceration of the oral mucosa [12]. No clinical trials have been published to establish the effectiveness of any particular treatment or prophylaxis [12]. The relationship between stress and psychiatric disorders and oral mucosa wounds and bites is highly relevant. Additionally, morsicatio, a form of self-inflicted physical trauma, is reported in young women and children [13,14]. Morsicatio involves chewing and friction of the mucous membranes due to stress or mental disturbance; lesions are most common on the buccal mucosa, tongue, and lips [13,14]. The dentist’s clinical response to oral mucosal wounds and stomatitis is to reduce irritation by adjusting orthodontic appliances and dentures and applying dexamethasone ointment. Patient options for self-care are limited to oral cleaning and dietary changes.

Bandages and patches that physically cover and protect wounds have been shown to prevent wound infections, maintain proper humidity for healing, and facilitate the healing process [15]. These effects are observed not only on the skin but also in the oral mucosal epithelium, and local gingival patches have been shown to promote a reduction in gingival inflammation [16]. Despite the potential needs of patients with oral mucosal wounds and stomatitis, the number of products and sales of liquid bandages for the oral mucosa is small, and a need exists to develop new liquid bandages specifically for the oral mucosa. The oral liquid bandage ORAPLA (FUJIFILM Toyama Chemical Co., Ltd., Tokyo, Japan) is a medical product that, when applied to the oral mucosa, reacts with saliva and other moisture to form a protective film that protects OM, stomatitis, and wounds caused by dentures and orthodontic appliances. This product contains a carboxyvinyl polymer (carbomer) as the base material, which has a proven track record as a water-soluble thickening agent in cosmetics and pharmaceuticals. Therefore, it is necessary to consider the acid erosion risk if the oral liquid bandage accidentally adheres to the teeth during application to the mucosa and whether secondary effects on the dental structure occur due to prolonged exposure. We set the null hypothesis that the acidity of the liquid bandage causes erosion. Our aim was to assess the acid erosion risk to the enamel and dentin when ORAPLA is deposited on teeth. We also compared the acid erosion risk at multiples of the maximum permissible duration of continuous use with those of over-the-counter drugs that have no reported health hazards. We find that the acid erosion risk due to accidental attachment to teeth is low, and in the human oral cavity with salivary buffering and remineralization, likely even lower.

## 2. Materials and Methods

### 2.1. Preparation of Enamel and Dentin Samples

A total of 45 bovine anterior mandibular teeth were collected from a slaughterhouse for this study. Five teeth were designated for each of the five experimental groups (*n* = 5/group). After removing the gingiva and cement, only the crown enamel and dentin were used in the experiment. The enamel and dentin blocks, measuring 1 cm × 1 cm × 1 cm, were prepared using a high-speed precision cutting machine (HS-45A, HEIWA technical Co., Ltd., Tokyo, Japan) and polished with water-resistant abrasive paper (#1000, #2000, and #4000). A window measuring 5 mm × 5 mm was created on the labial side of the enamel and dentin block using inlay wax. The methods of Vickers hardness testing and 3D laser microscopy measurements were taught by an instructor and calibrated between researchers. The Institutional Animal Ethics Committee at Tokyo Dental College granted approval for the animal study protocol, with approval number 230901 and an approval date of 1 April 2023. This study was reviewed and approved by the Tokyo Dental College conflict of interest committee (approval number 326, date of approval: 31 July 2023).

The oral liquid bandage ORAPLA (FUJIFILM Toyama Chemical Co., Ltd., Tokyo, Japan) is planned to be sold as a general medical device (notification number: 13B2X00129000001). Biological safety evaluations, including cytotoxicity tests (Nos. 410234 and 410241, Mouse L929cell, 63.7 mg/mL extraction solution, 7 days, in vitro), an adjuvant and patch test (No. 410238, Cavia porcellus Kwl:Hartley, skin application, 0.1 g/site 24 h × 3 times (primary sensitization), 0.2 g/site 48 h (secondary sensitization), 0.02 g/site 24 h (for challenge)), an oral mucosal irritation test (No. 410237, hamster Slc:Syrian, 0.1 g/site, once every hour, 4 times in total), and a systemic toxicity test (No. 410239, Rat Crlj:CD(SD), Oral intake of extract suspension (1835 and 917 mg/kg/day) for 28 days), were conducted by specialized institutions.

### 2.2. Acid Challenge Experiment with Artificial Salivary Circulation

**Figure 1:**
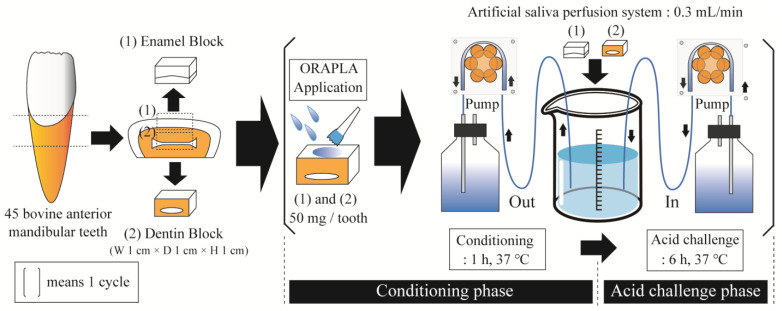
Overview of the acid challenge experiment. This experiment consists of a conditioning phase and an acid challenge phase, repeated, with one cycle in parentheses. Artificial saliva is continuously cycled through the peristaltic pump at a rate of 0.3 mL/min. Dotted lines in teeth means cutting line.

We created an apparatus in which enamel and dentin samples coated with ORAPLA were placed in a chamber in which artificial saliva was circulated to reproduce prolonged exposure conditions (Figure 1). The experiment comprised two phases: conditioning and acid challenge. The purpose of the conditioning phase was to wet and condition the tooth surface. Uncoated samples were immersed in artificial saliva for 1 h at 37 °C, and then ORAPLA was applied in an amount sufficient to cover the entire window surface (50–100 mg/tooth). During the acid challenge phase, the sample was placed in an artificial saliva annulus at 37 °C for 6 h. The conditioning and acid challenge phases were combined into one cycle (Figure 1). Four groups of enamel samples were established: (1) no ORAPLA application (control group), (2) the 3-cycle group (6 h exposure × 3 times = 18 h total), (3) the 6-cycle group (6 h exposure × 6 times = 36 h total), and (4) the 9-cycle group (6 h exposure × 9 times = 54 h total). Four groups of dentin samples were established: (1) no ORAPLA application (control group), (2) the 2-cycle group (6 h exposure × 2 times = 12 h total), (3) the 4-cycle group (6 h exposure × 4 times = 24 h total), (4) the 6-cycle group (6 h exposure × 6 times = 36 h total), and (5) the A-Corp group (6 h exposure × 6 times = 36 h total). The A-Corp dentin group was treated with an over-the-counter ointment used to treat inflammation and stomatitis of the oral cavity (The compositions of the A-Corp products are listed in Table 1). Like ORAPLA, A-Corp contains a carboxy vinyl polymer and is acidic in the oral environment; no reports of acid erosion or health problems have appeared since its release. The components of both ORAPLA and A-Corp products are listed in Table 1. In this study, A-Corp was utilized as a control drug for ORAPLA therapy. Five samples were prepared for each group (*n* = 5). To apply the experimental and control treatments on the same tooth surface, dental wax (Inlay Wax Soft, GC Co., Ltd. Tokyo, Japan) was applied to half of the mirror-finished enamel surface. An artificial saliva solution was prepared at the beginning of each cycle with a 0.02 M HEPES buffer (Sigma–Aldrich, St. Louis, MO, USA) that was supplemented with 3 mM Ca, 1.8 mM P, a pH of 7.3, and a degree of saturation of 10. The chamber was filled with 20 mL of artificial saliva per sample. The artificial saliva was cycled at a rate of 0.3 mL/min using a peristaltic pump throughout the conditioning and acid challenge phases. In addition, low-speed rotary stirring was continuously applied to homogenize the artificial saliva in the chamber.

### 2.3. 3D Laser Microscopy

The samples were dehydrated using an ascending ethanol series after wax removal. The step height profile was measured between the experimental and reference surfaces using a 3D laser microscope (LEXT OLS4000, Olympus, Tokyo, Japan) following the acid challenge. This experiment quantified the number of substantial defects caused by acid challenge on the teeth. The measurement area was set at 645 × 645 µm, and a photograph was taken of the boundary between the acid-demineralized experimental surface (ES) and the wax-protected reference surface (RS). Three-dimensional (3D) measurements were performed at five sites per sample, and the mean and standard deviation were calculated. The purpose of this analysis was to gauge the average roughness of the ES. To generate the roughness curve, wavelengths longer than 80 µm were excluded from the cross-sectional curve. The roughness curve was measured at five different locations per sample and placed at the boundary between the ES and RS. The count of significant defects and Sa were documented, and the mean and standard deviation (SD) were computed for each sample.

### 2.4. Micro-Vickers Hardness Measurement

The hardness of the samples was determined using a hardness tester (HMV-1; Shimadzu Corporation, Tokyo, Japan) after they had been dehydrated using an ethanol series. The indentation load was set at 0.49 N, and the time was set to 20 s. The change in hardness before and after the experiment (∆HV = RS − ES) was calculated to account for any variations between samples. Five locations on each sample were tested, and the mean and standard deviation were calculated for both the hardness values (HV) and the change in hardness values (∆HV).

### 2.5. Cross-Section and Surface Morphology by Scanning Electron Microscopy (SEM)

After completing the acid challenge, each sample group was cleaned with xylene and dehydrated using a series of increasing ethanol concentrations. Subsequently, the tooth surface was examined under a scanning electron microscope (SU6600, HITACHI, Tokyo, Japan) at a 15-kV accelerating voltage. Samples were embedded in polyester resin (Rigolac, Nisshin EM, Tokyo, Japan) to create polished sections, and the cross-sections were inspected.

### 2.6. Statistical Analysis

The average ± standard deviation of five samples was calculated to compare the sample groups. One-way analysis of variance (ANOVA) was used to determine the *p*-values, and the results were deemed significant at *p* < 0.05. When significance was determined by ANOVA, the Bonferroni test was used for post-hoc comparisons. The software program ORIGIN 2023 (Lightstone Corp, Tokyo, Japan) was utilized to create graphics and analyze data.

## 3. Results

### 3.1. Step Height Profiles after Acid Challenge: Enamel

**Figure 2:**
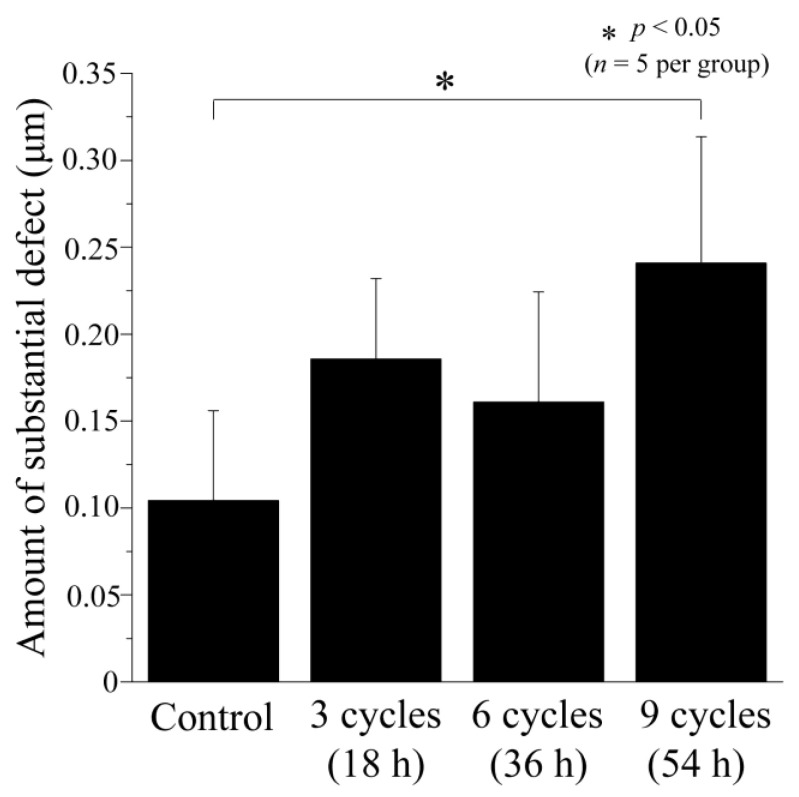
The amount of substantial defect in enamel was determined by measuring the step height profiles using a 3D laser microscope. This defect is defined as the step height difference between the ES and RS after an acid challenge for each enamel sample. Demineralization causes the enamel to exhibit substantial defects.

The enamel surface profiles after the acid challenge are depicted in Figure 2 through 3D laser microscopy measurements. The graph shows the step difference between the RS, which was coated with wax and did not demineralize, and the ES, which was the same tooth surface exposed to ORAPLA for an extended period (Figure 2). In the control group, no loss of mineral content was detected on the enamel surface, and a minor defect of 0.104 ± 0.052 μm was present. The 3-cycle group exhibited a larger defect amount of 0.186 ± 0.046 μm compared to the control group, but the distinction was not statistically significant (*p* > 0.05). The 6-cycle group also had a larger loss than the control group, 0.161 ± 0.063 μm, with no significant difference from the control group. The 9-cycle group had the largest defects of any group (0.241 ± 0.073 μm, *p* < 0.05 vs. control), but these were not significantly different from those of the 3- and 6-cycle groups.

### 3.2. Average Roughness (Sa) after Acid Challenge: Enamel

**Figure 3:**
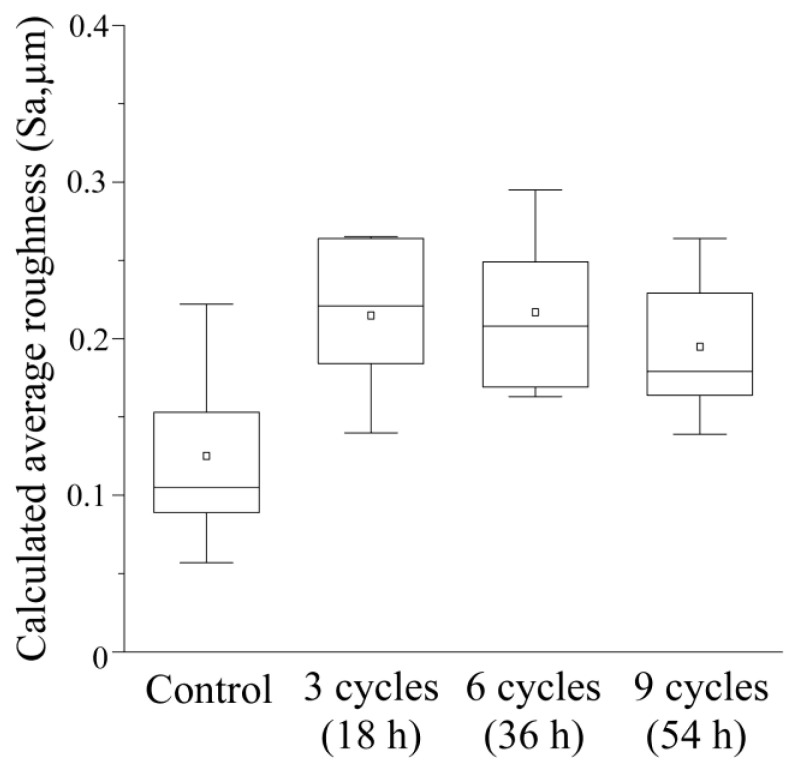
The enamel’s average roughness was determined following an acid challenge. The median value is represented by a horizontal line within a box, while the upper and lower boundaries represent the 25th and 75th percentiles, respectively. The white squares signify the mean values, and there were 5 samples per group.

Figure 3 displays a box plot of the groupwise results. The control group demonstrated no irregularities on the enamel surface, with a mean surface area (Sa) of 0.125 ± 0.057 μm and a median of 0.105 μm (interquartile range of 0.073–0.188). The 3-cycle group exhibited a mean Sa of 0.215 ± 0.048 μm, a median of 0.221 μm (0.162–0.265), and the 6-cycle group displayed the largest Sa among the four groups, with a mean of 0.217 ± 0.050 μm and a median of 0.208 μm (0.166–0.272). The 9-cycle group had a smaller Sa than the 3- and 6-cycle groups, with a mean of 0.195 ± 0.045 μm and a median of 0.179 μm (0.152–0.247). No significant differences were observed among the groups (*p* > 0.05).

### 3.3. Vickers Hardness and Its Changes after Acid Challenge: Enamel

**Figure 4:**
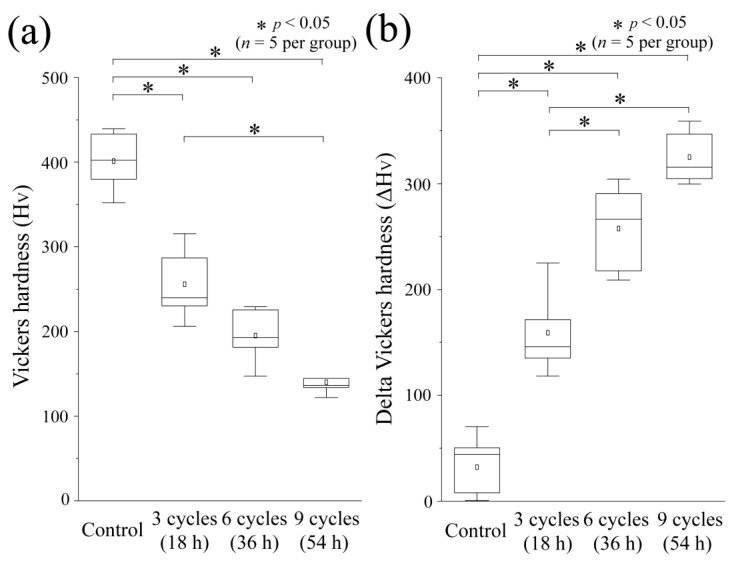
Vickers hardness measurements. (**a**) Boxplot of H_V_ values after acid challenge. Conventions are as in Figure 3. (**b**) Boxplot of ΔH_V_ values. ΔH_V_ is calculated as the change in HV before and after the experiment (∆HV = RS − ES).

The results of the Vickers hardness test for the ES in each group are depicted in Figure 4a. Among all groups, the control group had the highest mean Vickers hardness, which was 401.4 ± 32.72, with a median of 402.4 (365.9–436.3). There was a statistically significant difference between the control group’s mean Vickers hardness and that of all other groups (*p* < 0.05). In contrast to the control group, the 3-cycle group had a lower mean Vickers hardness of 255.7 ± 39.80 and a median of 240.0 (218.1–301.2). The 6-cycle group showed a smaller value, with a mean of 195.2 ± 30.36 and a median of 193.0 (164.1–227.5), which was comparable to the 3-cycle group. No significant differences were found between the 6-cycle and 3-cycle groups (*p* > 0.05). The Vickers hardness of the 9-cycle group was the lowest of any group, with a mean of 140.0 ± 13.49 and a median of 136.5 (128.0–153.7). A significant difference was observed between the 3-cycle and 9-cycle groups (*p* < 0.05).

Figure 4b displays the difference in Vickers hardness, which represents the change in hardness before and after the acid challenge. The control group displayed the least significant change in Vickers hardness, with a mean of 32.14 ± 39.85 and a median of 44.14 (2.015–60.31), and all other groups showed significant differences from each other (*p* < 0.05). The mean of the 3-cycle group was 159.1 ± 37.19, a median of 145.8 (126.7–198.2), demonstrating an increase compared with the control group. The 6-cycle group showed a significantly larger change amount than did the 3-cycle group, with a mean of 257.6 ± 38.25 and a median of 266.4 (213.3–297.5) (*p* < 0.05). The 9-cycle group exhibited the largest change in hardness: mean 325.2 ± 23.61 and median 315.5 (302.2–353.0). However, no significant difference was observed between the 6- and 9-cycle groups.

### 3.4. Step Height Profiles after Acid Challenge: Dentin

**Figure 5:**
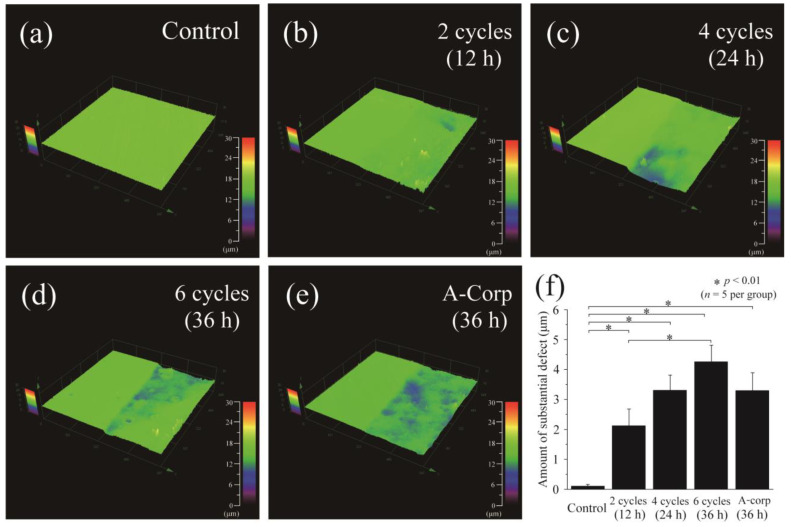
The 3D laser microscope was used to measure the step height profiles. The images in figures (**a**–**e**) show the boundary of the reference (RS) and experimental (ES) surfaces after an acid challenge. (**a**) control, (**b**) 2-cycle, (**c**) 4-cycle, (**d**) 6-cycle, and (**e**) A-Corp groups. The left side of each image shows the RS, which was protected by wax and not demineralized. The right side shows the ES, which has been exposed to ORAPLA for a long period. (**f**) Figure (**f**) shows the quantification of the substantial defects caused by demineralization. The results are presented for the control, 2-cycle, 4-cycle, 6-cycle, and A-Corp groups.

The diagram in Figure 5 depicts the outcomes of 3D laser microscopy measurements for the surface profiles of dentin after an acidic challenge. The left portion of Figure 5a–e represents the RS, which was shielded from demineralization and coated with wax, while the right portion displays the ES. The control group exhibited the least average defect size, with no observed demineralization and a value of 0.104 ± 0.059 μm. However, statistically significant differences were found between the control group and all other groups (*p* < 0.01; Figure 5a,f). In the 2-cycle group, the difference in height between the RS and ES was much larger than in the controls (2.123 ± 0.553 μm) (*p* < 0.01 vs. control; Figure 5b,f). The 4-cycle group also showed relatively large step values, with a mean of 3.302 ± 0.508 μm, comparable to the 2-cycle group. No significant differences were observed between the 2-cycle and 4-cycle groups (*p* > 0.05; Figure 5c,f). The 6-cycle group had the largest defect amount of all groups, 4.256 ± 0.553 μm (Figure 5d,f), being significantly larger than that of the 2-cycle group (*p* < 0.01). In the A-Corp group, the mean defect was 3.292 ± 0.595 μm, similar to that of the 4-cycle group. The A-Corp group showed no significant differences from the experimental groups and showed significantly greater defects than the controls (Figure 5e,f).

### 3.5. Average Roughness after Acid Challenge: Dentin

**Figure 6:**
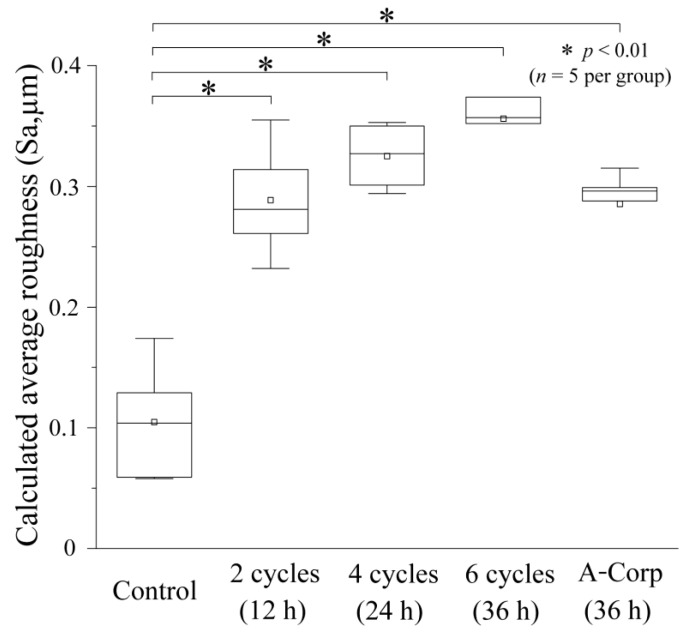
Calculated average roughness after acid challenge.

Figure 6 is a box plot representing the Sa outcomes for each group. The control group demonstrated the smallest Sa, with no demineralization observed, at a mean of 0.105 ± 0.044 μm, a median of 0.104 μm (0.058–0.152), and significant disparities were found when compared to all other groups (*p* < 0.01; Figure 6). In the 2-cycle group, the mean Sa was 0.289 ± 0.043 μm, with a median of 0.281 μm (0.247–0.335). The 4-cycle group had a mean Sa of 0.325 ± 0.024 μm and a median of 0.327 μm (0.298–0.352). The 6-cycle group exhibited the largest Sa among the five groups, with a mean of 0.356 ± 0.042 μm and a median of 0.357 μm (0.318–0.394). The A-Corp group displayed a mean Sa of 0.285 ± 0.029 μm, a median of 0.296 μm (0.259–0.307), and no significant disparities from the experimental groups, with a Sa that was significantly greater than that of the control (Figure 6).

### 3.6. Vickers Hardness and Its Changes after Acid Challenge: Dentin

**Figure 7:**
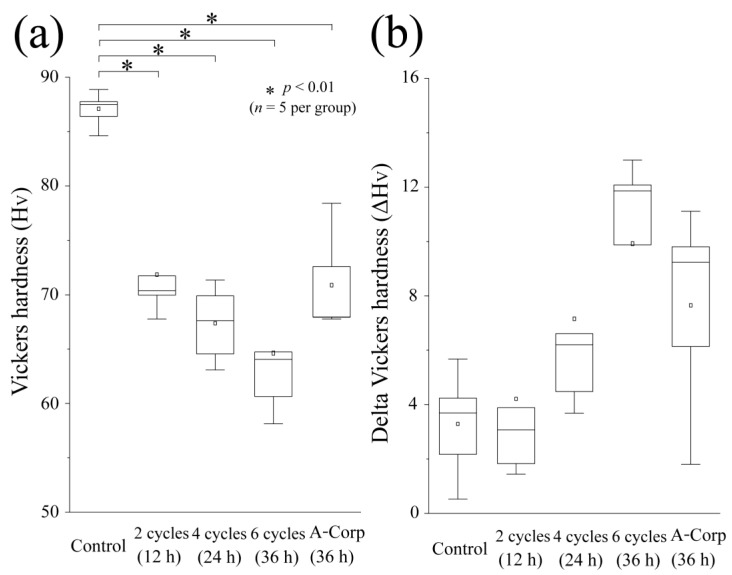
Micro-Vickers hardness measurements. (**a**) Boxplot of Vickers hardness (H_V_) values after acid challenge (*n* = 5; *, *p* < 0.01); (**b**) boxplot of ΔH_V_ values (difference in the H_V_ values between the RS and ES) (*n* = 5).

The graph in Figure 7a shows the Vickers microhardness test results for each group’s ES. The mean hardness of the control group was 87.03 ± 1.441, with a median of 87.50 (85.51–88.32), which was significantly higher than any other group (*p* < 0.01). The Vickers hardness of the 2-cycle group decreased compared with the control group, with a mean of 71.91 ± 4.113 and a median of 70.38 (68.87–75.73). The 4-cycle group still showed a smaller value, with a mean of 67.29 ± 3.112 and a median of 67.62 (63.82–70.62), comparable to the 2-cycle group. The hardness of the 6-cycle group was the lowest, with a mean of 64.61 ± 5.940 and a median of 64.06 (59.38–70.12). The mean hardness of the A-Corp group was 70.93 ± 4.16, with a median of 67.95 (67.86–75.50). No significant differences were found between the 6-cycle and A-Corp groups (*p* > 0.05).

Figure 7b shows ΔH_V_, which indicates the change in the Vickers hardness before and after the acid challenge. The control group exhibited the smallest change, with a mean of 3.261 ± 1.770 and a median of 3.690 (1.348–4.960). The mean ΔH_V_ of the 2-cycle group was 4.182 ± 3.367, median of 3.066 (1.635–7.286). The mean ΔH_V_ of the 4-cycle group was 7.135 ± 3.926, a median of 6.206 (4.087–10.65), demonstrating an increase compared with the control group. The 6-cycle group exhibited the greatest change, with a mean of 9.896 ± 3.753 and a median of 11.86 (6.275–12.53). The mean ΔH_V_ value of the A-Corp group was 7.620 ± 3.337, with a median of 9.240 (3.970–10.46); no significant differences from any experimental group were found (*p* > 0.05).

### 3.7. Surface SEM Observations after Acid Challenge: Dentin

**Figure 8:**
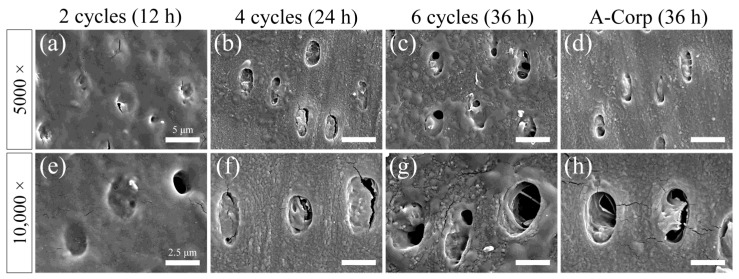
Scanning electron microscope (SEM) images of dentin surfaces after acid challenge. SEM image of the 2-cycle (**a**,**e**), 4-cycle (**b**,**f**), 6-cycle (**c**,**g**), and A-Corp (**d**,**h**) groups. (**a**–**d**) The scale bar is 5 μm. All images are recorded at 5000-fold magnification after Au–Pd deposition. (**e**–**h**) The scale bar is 2.5 μm. All images are recorded at 10,000-fold magnification after Au–Pd deposition.

A secondary electron image of the dentin surface after the acid challenge is shown in Figure 8. The surfaces of the 2-cycle group were uniform and smooth, with no significant irregularities due to acid damage. A high percentage of dentin tubules were closed, and when open, their diameters were small (Figure 8a,e). The 4-cycle group showed small irregularities due to acid damage, and some dentin around the tubules showed roughness due to acid demineralization. A greater percentage of dentinal tubules were closed than open, but the number of open tubules in the 4-cycle group was higher than in the 2-cycle group (Figure 8b,f). The 6-cycle group showed small irregularities due to acid damage, and some peritubular dentin showed more advanced roughness than the 4-cycle group (Figure 8c,g). The percentage of closed dentinal tubules was approximately 50–60%, with even more open tubules than in the 4-cycle group. However, even when a tubule opening was observed, the diameter of the opening was often small, with 60–80% of the cross-sectional area of the tubule occluded (Figure 8c,g). The A-Corp group showed small irregularities due to acid damage, and the appearance of the dentin around the tubules was similar to that in the 4-cycle group. The percentage of closed dentinal tubules was about 60–70%, and the number of open tubules was similar to that in the 4-cycle group. Even when tubular openings were observed, they were often small in diameter, and 60–80% of the cross-sectional area of the tubules was occluded (Figure 8d,h).

### 3.8. Cross-Sectional SEM Observations after Acid Challenge: Dentin

**Figure 9:**
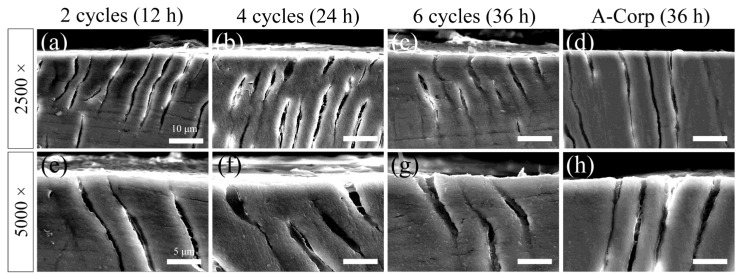
SEM images of dentin cross-sections after acid challenge. Cross-sectional SEM image of the 2-cycle (**a**,**e**), 4-cycle (**b**,**f**), 6-cycle (**c**,**g**), and A-Corp (**d**,**h**) groups. (**a**–**d**) The scale bar is 10 μm (2500-fold magnification). (**e**–**h**) The scale bar is 5 μm (5000-fold magnification). (**a**–**h**) All images are recorded after carbon evaporation onto the sample.

A reflected electron image of the cross-sectioned ES after the acid challenge is presented in Figure 9. Cross-sectional images of the 2-cycle group showed no lateral enlargement of the opening or depth of dentin tubules. Moreover, we observed no dentin demineralization attributable to acid leaching of calcium (Figure 9a,e). Similarly to the 2-cycle group, the 4-cycle group did not show lateral enlargement of the openings, increases in the depth of the dentin tubules, or demineralization, but a funnel-shaped enlargement was observed at the opening of some tubules (Figure 9b,f). The 6-cycle group did not differ from the 4-cycle group in terms of the dentin tubules or dentin, and we observed no evidence of acid erosion (Figure 9c,g). We observed no structural differences between the A-Corp group and the 6-cycle group (Figure 9g,h).

## 4. Discussion

### 4.1. Assessment of Dental Erosion Risk: Enamel

Long-term enamel exposure to ORAPLA for up to six cycles (36 h) did not cause substantial loss, and the effect on the internal structure, accessed as Vickers hardness, was mild and within the range of healthy teeth, indicating a low erosion risk. We established a control group as a model of the teeth adjacent to the tooth with ORAPLA attached (3-, 6-, and 9-cycle groups). The control group was placed in the same chamber and artificial saliva as the exposed group for 54 h (the maximum exposure time) and was therefore affected by ORAPLA and components dissolved from the exposed group in the artificial saliva. The control group showed no evidence of acid erosion after 54 h, with no substantial defects, increases in surface roughness, or decreases in Vickers hardness (Figure 2, Figure 3 and Figure 4). These results suggest that when ORAPLA is applied to the mucosa under normal conditions, oral enamel is not subject to acid erosion, even after prolonged, continuous use. The 3-cycle group, a group in which enamel was coated directly, showed no significant differences from the control group in terms of substantial defects, surface roughness, or Vickers hardness, which were within the ranges of sound teeth (Figure 2, Figure 3 and Figure 4). The Vickers hardness (H_V_) of sound enamel is approximately 270–366 [17]. The mean Vickers hardness of the 3-cycle group was almost the same as that of the sound teeth, considering individual differences (Figure 4a). The 6-cycle group showed no significant differences from the control group in substantial defects or surface roughness, but the Vickers hardness decreased (Figure 2, Figure 3 and Figure 4). The mean Vickers hardness of the 6-cycle group was below the range of healthy teeth, suggesting that mild acid damage to the internal structure of the enamel had occurred (Figure 4a). The decrease in Vickers hardness with increasing exposure time was also observed in ΔH_V_, and significant differences were observed between the 3-cycle and 6-cycle groups (Figure 4b). In the 9-cycle group, where the exposure time was increased to 54 h, we observed no change in surface roughness, but a small substantial defect was observed, and Vickers hardness decreased, indicating mild acid corrosion (Figure 2, Figure 3 and Figure 4).

Previously, the enamel substantial defect amount observed following a 60-min exposure to acidic liquids was estimated by the formula “Enamel loss (μm) = 6.676 − 1.726 pH + 0.233 TA (titratable acidity) [18,19]. The commercial carbonated beverage (a cola) used to obtain this equation produced a defect of 3.05 ± 0.74 μm [18,19]. A confocal laser scanning microscope study of enamel exposed to several commercial beverages (pH 2.42–3.46) for 60 min also observed 5–10 μm defects [19]. These studies are not comparable to ours because they employed experimental systems in which artificial saliva was not present. However, the fact that the 9-cycle group had a loss of 0.241 ± 0.073 μm even after 54 h of continuous exposure, the largest observed here, suggests that the damage to enamel caused by ORAPLA was much less severe than that caused by commercial carbonated beverages. Daily eating and drinking are considered more dangerous factors for acid erosion than are drugs or medical procedures [20,21,22]. A cohort study of the relationship of possible etiological factors to tooth erosion in 1753 children found a significant positive association between fruit juice consumption (odds ratio [OR] = 1.42) or carbonated beverage consumption (OR = 1.59–2.52) and the occurrence of acid erosion following medical procedures [20,21,22]. The maximum continuous use time for normal use listed in the ORAPLA attachment is 6 h; this time limit was used here to set the scale of the experimental exposure times, which were integer multiples of 6 h. In the oral cavity, it is unlikely that ORAPLA will continuously adhere for longer than 6 h, owing to physical abrasion and water flow caused by saliva swallowing, eating, drinking, and talking. In addition, saliva has a powerful carbonic acid buffering capacity and the ability to remineralize eroded teeth [23,24,25]. Long-term enamel exposure to ORAPLA for up to 36 h (6 h exposure × 6 times), which is six times longer than the normal use limit, did not cause substantial defects, and the damage to the internal structure was mild. Continuous exposure for 54 h, which is nine times the normal use limit, was associated with substantial minute defects. However, under conditions of salivary buffering and remineralization, it is unlikely that ORAPLA accidentally adhering to the enamel will induce acid erosion.

### 4.2. Risk Assessment of Dental Erosion: Dentin

Dentin contains a higher collagen percentage than enamel and has a higher critical pH (6.0–6.2 vs. 5.5), making it more susceptible to acid erosion than enamel [26,27]. In dentin, the number of substantial defects, surface roughness, and Vickers hardness tended to decrease with increasing ORAPLA exposure time (Figure 5, Figure 6 and Figure 7). However, no measurement showed significant differences from those of the control A-Corp group at the maximum exposure time, 36 h. The control group showed no evidence of substantial defects, increased surface roughness, or decreased Vickers hardness, and no evidence of acid erosion after incubation in the same simulated oral cavity as the exposed groups (2-, 4-, and 6-cycle) (Figure 5, Figure 6 and Figure 7). These results suggest that, under normal usage with application to mucous membranes only, dentin is not subject to acid erosion, even after prolonged continuous use. The 2-cycle group showed significant minor substantial defects and increased surface roughness compared with the control group, but the Vickers hardness was within the range of sound dentin (Figure 5, Figure 6 and Figure 7) [17]. The change in Vickers hardness was not significantly different from that of the control group, and the SEM findings showed no enlargement or damage to the dentin tubule openings (Figure 7b). The 4-cycle and 6-cycle groups showed an increasing trend of substantial defects and surface roughness with increasing exposure time, but we observed no statistically significant difference between the groups (Figure 7). Surface and cross-sectional SEM findings in the 4-cycle and 6-cycle groups revealed openings and funnel-shaped widening of some dentin tubules; however, most of the tubules were closed, suggesting that acid erosion and dentin hypersensitivity were unlikely to be induced (Figure 8 and Figure 9). Here, A-Corp, a commercially available drug with no safety issues, was used as a control drug. No significant differences were observed between the ORAPLA application 6-cycle group and the A-Corp group in any measure, suggesting no difference in acid erosion risk. The A-Corp group showed substantial defects, increased surface roughness, and decreased Vickers hardness, similar to observations of the ORAPLA 2-cycle and 4-cycle groups (Figure 5, Figure 6 and Figure 7). Therefore, based on cariological, histological, scientific, and engineering findings, the risk of acid caries from continuous exposure to ORAPLA within 36 h is considered low and comparable to that of over-the-counter drugs. The surface SEM after a 36-h exposure to ORAPLA showed no difference from that of the over-the-counter drug, with extremely mild enlargement or opening of the dentin tubules. Therefore, it is unlikely that subjective symptoms such as hypersensitivity would be observed in practice.

### 4.3. Limitations and Generalizability to the Human Oral Environment

(1) This study was conducted in vitro under conditions differing from a real human oral cavity. Artificial saliva, stirrers, and fluid flow were used to approximate the oral environment. The artificial saliva included a simple buffering capacity and calcium and phosphorus saturation; however, salivary proteins, pellicles, and oral bacteria were not present [28]. The artificial saliva was based on HEPES buffer and followed a recipe described previously [29]. HEPES has a pKa of 7.55 and a pH range of 6.8–8.2 and has been used as a buffer in biochemical fields such as cell culture and tissue storage [29]. However, human saliva is composed of a carbonic acid/bicarbonate buffer system more powerful than HEPES, accounting for 85–95% of the total buffering capacity [23,24,25,30]. Therefore, in the human oral cavity containing saliva, demineralization is likely to be inhibited more strongly than would be observed in our model. In addition, stimulated saliva secreted at mealtime has a stronger buffering effect than resting saliva because it is secreted in larger volumes [23,24,25,30]. This suggests that demineralization is inhibited to a greater extent during eating or snacking, while ORAPLA adheres to teeth.

(2) Physical stimulation from eating, drinking, and cheek vibrations occurring during conversation are also expected to mitigate the effects of ORAPLA attachment to teeth. Here, the exposure time per cycle was set at 6 h based on the assumption that the product would be reapplied after each of three meals over 18 h per day, excluding sleeping time. However, physical stimuli and degradation due to hydration of the gelled ORAPLA deposit may reduce the actual exposure time in the oral cavity.

(3) Most crucially, a difference in the compositions of human and artificial saliva related to demineralization not modeled here is the presence or absence of fluoride. Application of high fluoride concentrations can reduce the acid erosion of dentin by approximately half or more [31,32,33]. Fluoride-containing toothpastes account for more than 90% of the toothpaste market, and children and adults alike are exposed to 500–1500 ppm of fluoride ions multiple times a day through daily self-care [34,35,36]. Although fluoride exposure was not assessed here because bovine teeth were used, in human teeth, approximately 1800 ppm of fluoride ions are present on the tooth surface and inhibit demineralization [37]. The fluoride concentration in human saliva is 0.05–0.25 ppm but increases to 1–3 ppm after the use of a fluoride-containing dentifrice [36,38]. Fluoride ions react with calcium in saliva to form calcium fluoride on the tooth surface, thereby increasing the acid resistance of the enamel and dentin and inhibiting demineralization [32,34,36]. There is evidence that brushing using fluoride toothpaste before eating and drinking acidic food/drink may reduce the effects of erosion [32,34,36]. These findings suggest that in the human oral environment with fluoride dentifrice use, the acid erosion risk posed by ORAPLA is lower than that observed here. The amount of acid produced by the carboxyl vinyl polymer in ORAPLA is very small, and the chemical reaction is completed in a short time. Therefore, it is unlikely that the gel will remain acidic for a long time due to the neutralization reaction in the human oral cavity. In addition, in this experiment, a sufficiently large amount of product was applied to the teeth to cover the window area, but in the oral cavity, only a small amount will be transferred from the mucosa to the teeth because only a portion of the mucosa is in contact with the teeth. Given the presence of fluoride in saliva and dentin and a calcium-supersaturated environment, it is unlikely that small amounts of acid will dissolve calcium fluoride from teeth to cause severe demineralization.

(4) One factor not addressed here that affects both the adhesion time and potential demineralization under the liquid bandage is individual differences in salivary secretion. Saliva secretion averages 0.3 mL/min for resting saliva and 1.5–2.0 mL/min for stimulated saliva, with very large individual differences observed [23,24,25]. In addition, saliva suppression is a side effect of many types of drugs, and dry mouth and OM are problems for patients taking multiple drugs simultaneously for diseases such as hypertension and diabetes, as well as for older patients [25,39,40]. The artificial saliva composition used here replicated general saliva composition and saturation and did not assume xerostomia. In xerostomia, decreased salivary secretion weakens the buffering capacity of the saliva and reduces the physical exfoliation of ORAPLA, increasing the acid erosion risk [41]. The effect of salivary secretion rate on the length of time the liquid bandage is maintained in the oral cavity is also unclear.

(5) Anatomical tooth shapes were not reproduced in our model. Salivary washout is greater on smooth surfaces but significantly lower on adjacent and interdental surfaces [42]. Separate risks should be considered for areas where the gelatinized liquid bandage is likely to remain in place versus areas where it is likely to be washed away.

(6) We considered the control group to be synonymous with negative controls. In the control group of this study, the liquid bandage was not applied and was only present in artificial saliva; therefore, it was not demineralized by acid. Although we previously discussed positive controls, there have been no previous studies in which acid erosion occurred with liquid bandages, and we were unable to find any appropriate reports. Liquid acids, such as lactic acid and hydrochloric acid, used in acid erosion experiments were not used because their viscosity and dynamics are different from those of liquid bandages.

### 4.4. Future Directions

In vivo and in vitro experiments should be conducted in the human oral environment to investigate the effect of individual differences on the acid corrosion risk posed by ORAPLA. Pellicles derived from salivary proteins exist on the tooth surfaces in the human oral cavity and act as a defense against acids, so it is desirable to devise an experimental system that takes pellicles into consideration.

### 4.5. Benefits and Clinical Dental Applications of Oral Liquid Bandages

For patients, pain relief after dental work has a higher priority than amelioration of the targeted disease, and the impact of oral liquid bandage use on the improvement of patients’ quality of life may be significant. In studies investigating the connection between denture stomatitis, traumatic ulcers, and various factors such as age, gender, education, smoking status, denture age, cleaning habits, and wearing behavior, 35.8% of patients exhibited stomatitis, while 29% had traumatic ulcers [43]. Research indicates that denture stomatitis affects approximately 15–70% of denture wearers, and ill-fitting dentures contribute to increased mucosal trauma [8]. In clinical dentistry, Vaseline (CAS:8009-03-8, FUJIFILM Wako, Tokyo, Japan) or Cocoa Butter (GC Dental Corp., Tokyo, Japan) is applied to patients with mouth-angle sores to reduce pain when opening the mouth during examination and to protect the wound. However, Vaseline does not provide long-term protection. Currently, few options are available for orally indicated adhesive bandages for mucosal protection, and the need for liquid bandages is potentially high.

We find that the acid erosion risk to either enamel or dentin within the recommended ORAPLA use times is small. Furthermore, regular professional care, such as mechanical tooth cleaning and fluoride application at the dentist’s office, and appropriate self-care, such as the use of fluoride-containing toothpaste during oral cleaning, will greatly minimize the acid erosion risk. However, inadequate self-care should be addressed by shortening the follow-up period for preventive and periodic checkups. OM caused by biting or oral diseases cannot be addressed by this product and requires close examination by a dentist. This product is intended for self-care only and should only be applied after a regular dental checkup.

## 5. Conclusions

The acid erosion potential of ORAPLA was found to be low, with no significant acid corrosion findings in enamel after up to nine times the manufacturer-recommended upper limit of 6 h of normal use and in dentin after six times this limit. A comparison of ORAPLA with a similar control drug with no reported health hazards revealed no significant differences in any measures used here. Thus, the acid erosion risk due to accidental ORAPLA attachment to teeth demonstrated here is low and is likely to be even lower in the human oral cavity, where salivary buffering and remineralization are present.

## Figures and Tables

**Table 1:** Composition of medical products used in this study cited.

Product	pH	Amount Use	Composition
ORAPLA (Experimental drug)	4.67	300 mg	Carboxyvinyl polymer (acidic causative agent), Gelled hydrocarbon, Sodium alginate, Aluminium lactate
A-Corp (Anonymous, over-the-counter drug)	5.58	300 mg	Carboxyvinyl polymer (acidic causative agent), Gelled hydrocarbon, Xylitol, Hypromellose, L-Menthol

## Data Availability

All data are contained within the article.
